# High-resolution medical image reconstruction based on residual neural network for diagnosis of cerebral aneurysm

**DOI:** 10.3389/fcvm.2022.1013031

**Published:** 2022-10-19

**Authors:** Bo Wang, Xin Liao, Yong Ni, Li Zhang, Jinxin Liang, Jiatang Wang, Yongmao Liu, Xianyue Sun, Yikuan Ou, Qinning Wu, Lei Shi, Zhixiong Yang, Lin Lan

**Affiliations:** Department of Neurosurgery, The Second Affiliated Hospital of Guizhou Medical University, Kaili, China

**Keywords:** ResNet, CTA, brain aneurysm, medical image, super-resolution

## Abstract

**Objective:**

Cerebral aneurysms are classified as severe cerebrovascular diseases due to hidden and critical onset, which seriously threaten life and health. An effective strategy to control intracranial aneurysms is the regular diagnosis and timely treatment by CT angiography (CTA) imaging technology. However, unpredictable patient movements make it challenging to capture sub-millimeter-level ultra-high resolution images in a CTA scan. In order to improve the doctor's judgment, it is necessary to improve the clarity of the cerebral aneurysm medical image algorithm.

**Methods:**

This paper mainly focuses on researching a three-dimensional medical image super-resolution algorithm applied to cerebral aneurysms. Although some scholars have proposed super-resolution reconstruction methods, there are problems such as poor effect and too much reconstruction time. Therefore, this paper designs a lightweight super-resolution network based on a residual neural network. The residual block structure removes the B.N. layer, which can effectively solve the gradient problem. Considering the high-resolution reconstruction needs to take the complete image as the research object and the fidelity of information, this paper selects the channel domain attention mechanism to improve the performance of the residual neural network.

**Results:**

The new data set of cerebral aneurysms in this paper was obtained by CTA imaging technology of patients in the Department of neurosurgery, the second affiliated of Guizhou Medical University Hospital. The proposed model was evaluated from objective evaluation, model effect, model performance, and detection comparison. On the brain aneurysm data set, we tested the PSNR and SSIM values of 2 and 4 magnification factors, and the scores of our method were 33.01, 28.39, 33.06, and 28.41, respectively, which were better than those of the traditional SRCNN, ESPCN and FSRCNN. Subsequently, the model is applied to practice in this paper, and the effect, performance index and diagnosis of auxiliary doctors are obtained. The experimental results show that the high-resolution image reconstruction model based on the residual neural network designed in this paper plays a more influential role than other image classification methods. This method has higher robustness, accuracy and intuition.

**Conclusion:**

With the wide application of CTA images in the clinical diagnosis of cerebral aneurysms and the increasing number of application samples, this method is expected to become an additional diagnostic tool that can effectively improve the diagnostic accuracy of cerebral aneurysms.

## Introduction

### Brain aneurysms and hazards

Intracranial aneurysms are cerebral hemangioma-like protrusions caused by abnormal local blood vessel morphology changes. The etiology of aneurysms is not clear. Genetic factors include: the thickness of the cerebral artery wall being about 1 % thinner than that of other parts of the same diameter or so, and lack of surrounding tissue support, the blood flow is significant, especially at the bifurcation is more vulnerable to the impact of blood flow; acquired factors include infection, trauma, tumor and atherosclerosis; most of them are genetic factors (1). The formation mechanism of the intracranial aneurysm is the stress damage to blood flow to the arterial wall. Due to the change in blood flow, a part of the arterial wall protrudes outward, forming a permanent local expansion. The magnitude of the stress is usually related to the velocity and angle of the blood vessel. Intracranial aneurysms are classified as severe cerebrovascular diseases due to their insidious onset, complex pathogenesis and acute onset (2).

Aneurysmal subarachnoid hemorrhage (aSAH) has consistently ranked among the cerebrovascular diseases that seriously endanger human health due to its insidious onset and complex pathogenesis. According to incomplete statistics, the incidence of bleeding has also remained high, accounting for about 85%. The most common symptom of aSAH is sudden severe headache, often described as the most severe and severe headache of the lifetime, with vomiting, nausea, photophobia, neck stiffness, transient loss of consciousness, or focal neurological deficits, such as cranial nerve palsy (3). Fortunately, 2– 8 weeks before some aneurysms rupture, patients may have “warning” symptoms in advance. Some patients are self-reported to have very mild cold-like headaches or mild nausea and vomiting that lasted for several days. It is difficult to be associated with severe intracranial hemorrhage. If relevant examinations are carried out in this period for differential diagnosis and symptomatic and supportive treatment, the occurrence of fatal hemorrhage can be largely avoided.

At present, the clinical symptoms of SAH patients are mainly determined by the Hunt-Hess classification, Glasgow Coma Scale (GCS), World Federation of Neurological Surgeons (WFNS) and prognostic scores on admission for aneurysmal subarachnoid hemorrhage (prognosis on the admission of aneurysmal subarachnoid hemorrhage, PAASH) (4). This study used the Hunt-Hess classification according to the condition at the time of admission: grade I: asymptomatic, or mild headache, mild neck stiffness; grade II: moderate to severe headache, neck stiffness or cranial nerve palsy; grade III I: lethargy or confusion, mild focal neurological impairment; grade IV: coma, moderate to moderate hemiplegia; grade V: deep coma, cerebral rigidity, moribund state. Regardless of the typical clinical symptoms of the disease, once the tumor ruptures and bleeds, there is a high risk of death and disability. Therefore, early diagnosis and symptomatic surgical treatment can effectively prevent the recurrence of intracranial aneurysms and reduce the rebleeding of intracranial aneurysms, thereby further improving the survival rate and quality of life of patients (5).

### CTA imaging technology

CTA is convenient, fast and straightforward to operate and can display the three-dimensional structure of cerebral blood vessels, which has a specific guiding role in detecting and detecting aneurysms (6). CTA can also observe and restore the internal conditions of blood vessels from different directions and angles, maximizing the accurate display of the three-dimensional anatomy of the aneurysm body, neck and parent artery from multiple angles and maximizing the natural restoration of multiple angles (7). The shape, size, and adjacent relationship of the tumor body provide the anatomical information of various aneurysms such as tumor wall calcification and intratumoral thrombus to the greatest extent accurately and display brain tissue lesions. The preservation of V.R. images of skull base provides essential reference information for clinical selection of surgical approach and provides help for the selection of surgical approach (8). The V.R. image is closer to the actual anatomical structure. It can display the diameter of the aneurysm, the width of the aneurysm, the shape of the aneurysm and the protruding direction of the aneurysm. Ninety-three percent of intracranial aneurysms occur in the circle of Willis and its branches, bifurcations and bends. CTA can display the course and variation of the circle of Willis and its branches, as well as the vertebral-basilar artery system and the circle of Willis. It has obvious advantages in observing the blood supply of the intracranial arteries, the degree of collateral circulation opening, and comparing the blood vessels on both sides. CTA is a non-invasive examination technique that can effectively reduce the occurrence of vasospasm, plaque and thrombus shedding caused by invasive surgery (9).

### High-resolution medical image reconstruction technology

In most digital imaging applications, images are often used and viewed by people, especially in the eyes of doctors, who prefer to choose high-quality, high-resolution images as diagnostic references (10). Improving the resolution of an image is a widely studied problem in computer vision. Its purpose is to generate high-resolution images from one or more low-resolution images. Super-resolution reconstruction (S.R.) algorithms aim to generate finer details than the sampling grid of a given imaging device by increasing the number of pixels per unit area in an image. S.R. is a well-known ill-posed inverse problem, recovering high-resolution images from low-resolution images (often affected by noise, motion blur, cerebral aneurysms, optical distortion, etc.) (11). S.R. techniques can be applied in many scenarios, where multiple frames of images of a single scene can be obtained (for example, a single camera can obtain multiple images of the same object), and various images of the scene can be obtained from many sources, such as multiple cameras from different Position captures a single scene. S.R. has applications in various fields, such as satellite imaging, remote sensing imaging, security and surveillance where it may be necessary to zoom in on specific points of interest in a scene (e.g., zoom in on criminal faces or license plate numbers), in computers that can improve pattern recognition performance The field of vision, as well as other fields such as facial image analysis, text image analysis, biometric recognition, fingerprint image enhancement, etc. S.R. is especially important in medical imaging, where more detailed image detail information is required, and high-resolution medical images can help doctors make correct diagnoses. For example, in computed tomography (C.T.) for diagnosis, the resulting low-resolution images have lower pixel density and provide minor detail due to various uncertainties, which significantly reduces the efficiency of doctor visits.

However, the imaging acquisition device or imaging acquisition process consisting of imaging sensors limits the resolution of the images. In theory, the higher the density of sensors in a digital imaging device, the higher-resolution images it is possible to produce. In fact, due to product cost considerations and the limitations of today's integrated circuits, increasing the number of sensors on a fixed area of a device is not an easy task. Post-processing methods such as super-resolution reconstruction can break through traditional physical limitations and improve image resolution. With the increase in human production and life, the demand for high-resolution images increases (12). With the rise of artificial intelligence technology and the rapid development of Graphics Processing Unit (GPU), deep learning to tap various complex image processing tasks has excellent potential and has become the favorite of many researchers. Deep learning mainly simulates human thinking and analysis by constructing deep neural networks, excavating potential laws between samples, extracting useful feature information, and assisting human beings in various data processing tasks.

In today's high incidence of various diseases such as cancer, high-resolution medical imaging technology is an essential basis for containing various information, which can accurately determine the location and direction of the disease for doctors to understand the disease more comprehensively assist doctors. Medication treatment. This paper uses deep learning technology to analyze various medical images in detail. The methods for super-resolution reconstruction of medical images in three different situations are discussed, effectively avoiding the defects of artificial extraction of data features in traditional methods. Compared with the hardware method, it has excellent advantages. To sum up, super-resolution techniques to improve the quality of medical images have significant research value for helping medical diagnosis (13).

## Related work

### Interpolation-based super-resolution reconstruction

Interpolation-based super-resolution reconstruction methods are implemented in the spatial domain, and most of the commonly used interpolation algorithms belong to non-uniform interpolation (14). The image after super-resolution reconstruction based on the interpolation algorithm is based on the most intuitive pixel information. The method is based on simple image prior information, so the image information used by this method is limited when we reconstruct the image. Generally, the magnification should not be too large, generally not more than twice the magnification. Otherwise, the reconstructed image will be prone to be over-smoothed at the edges or discontinuous points of the image. The nearest neighbor interpolation method, bilinear interpolation, and bicubic interpolation algorithms are the most commonly used interpolation algorithms. The following is a brief introduction to the three traditional interpolation algorithms.

The idea of this method is to restore the lost pixels by using the gray value of the known pixels around the point to be interpolated, calculate the pixels of the point to be found, and realize the upsampling and zooming operation of the image, such as nearest-neighbor interpolation, bilinear interpolation method and bicubic interpolation (15).

However, due to the shortcomings of this kind of algorithm, such as being significantly affected by noise and artificial artifacts in reconstructed images, they are primarily used in situations where the resolution requirements of images are not exceptionally high.

### Reconstruction-based super-resolution reconstruction

**Figure 1:**
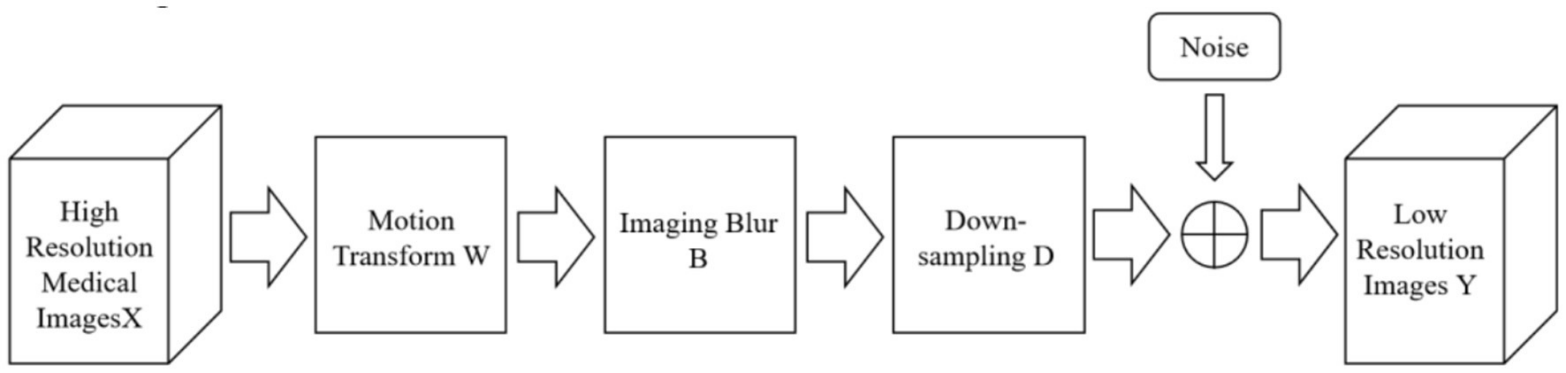
Reconstruction-based super-resolution reconstruction step diagram.

The theoretical basis of reconstruction-based methods is the degradation model of images. The reconstruction-based method uses the prior knowledge of the image as a constraint to realize the super-resolution reconstruction of the image, such as smoothing prior, reconstruction prior and so on. Standard reconstruction methods mainly include the iterative back-projection method, convex set projection method, and maximum a posteriori probability estimation (16). The reconstruction steps are shown in Figure 1.

The super-resolution reconstruction can be regarded as the inverse process of the image degradation process. The high-frequency information lost in the degradation process is added to the image reconstruction process. The image super-resolution reconstruction problem is finally transformed into the inverse solution of the image degradation model.

Different image acquisition devices often use different imaging technologies to acquire images. However, the acquired images usually have different degrees of degradation, affected by device accuracy and environmental factors. In order to reconstruct a super-resolution image from the degraded low- resolution image, the degradation process of the image needs to be modeled first. In the field of medical imaging studied in this paper, the main factors that cause the degradation of imaging quality are insufficient radiation dose, too low sampling frequency, sampling blur caused by patient motion, and equipment noise (17). Its degradation process can be approximated by Equation (1), and the corresponding mathematical model is:


(1) $$\begin{array}{l} {\text{I}}_{\text{LR}}={\text{G}}_{\text{blur}}\left({\text{I}}_{\text{HR}}\right){↓}_{\text{s}}+{\text{n}}_{\sigma } \end{array}$$

Among them, *I*_*LR*_ is the degraded low-resolution image, which *I*_*HR*_ is the original high-resolution image, which *G*_*blur*_ represents the blur function, usually Gaussian blur, which represents the ↓_*s*_ downsampling method with a *n*_σ_ downsampling coefficient of, which represents additive noise.

Based on the above definition of the image degradation model, the super-resolution reconstruction algorithm can be defined as Equation (2):


(2) $$\begin{array}{l} {\text{I}}_{\text{SR}}={\text{F}}_{\text{s}}\left(\text{P}\left({\text{I}}_{\text{LR}}\right)\right) \end{array}$$

*P*(·) represents the preprocessing process for low-resolution images, such as upsampling, filtering, noise reduction, etc. In the algorithm designed in this paper, the preprocessing operation is directly removed, making the algorithm more concise; *F*_*s*_(·) represents the upsampling coefficient. Rebuild the model for super-resolution.*s*.

### Learning-based super-resolution reconstruction

The basic idea is to learn the mapping relationship between high-resolution and low-resolution images. The learning process is divided into training and guided reconstruction, based on dictionary learning and based on the neural network (18).

Compared with interpolation-based and reconstruction-based algorithms, learning-based algorithms are more in line with the current research trend of intellectual development. Learning-based super-resolution reconstruction algorithms used in medical images are mainly divided into general and deep learning algorithms.

SRCNN is the first attempt to apply deep learning to the field of super-resolution reconstruction. It is the first application of deep learning in this field. He is a deep learning method for single image super-resolution (S.R.) (19). End-to-end learning is achieved by directly learning the functional relationship between low-resolution and high-resolution images. In traditional sparse coding-based super-resolution reconstruction methods, for each component, each step requires a specific method to be processed separately, which implements a joint optimization of all neural network layers. Moreover, because the structure is relatively shallow, the structure is light, the speed of reconstruction efficiency is better than the traditional method, and the quality of the reconstructed image effect is also greatly improved.

**Figure 2:**
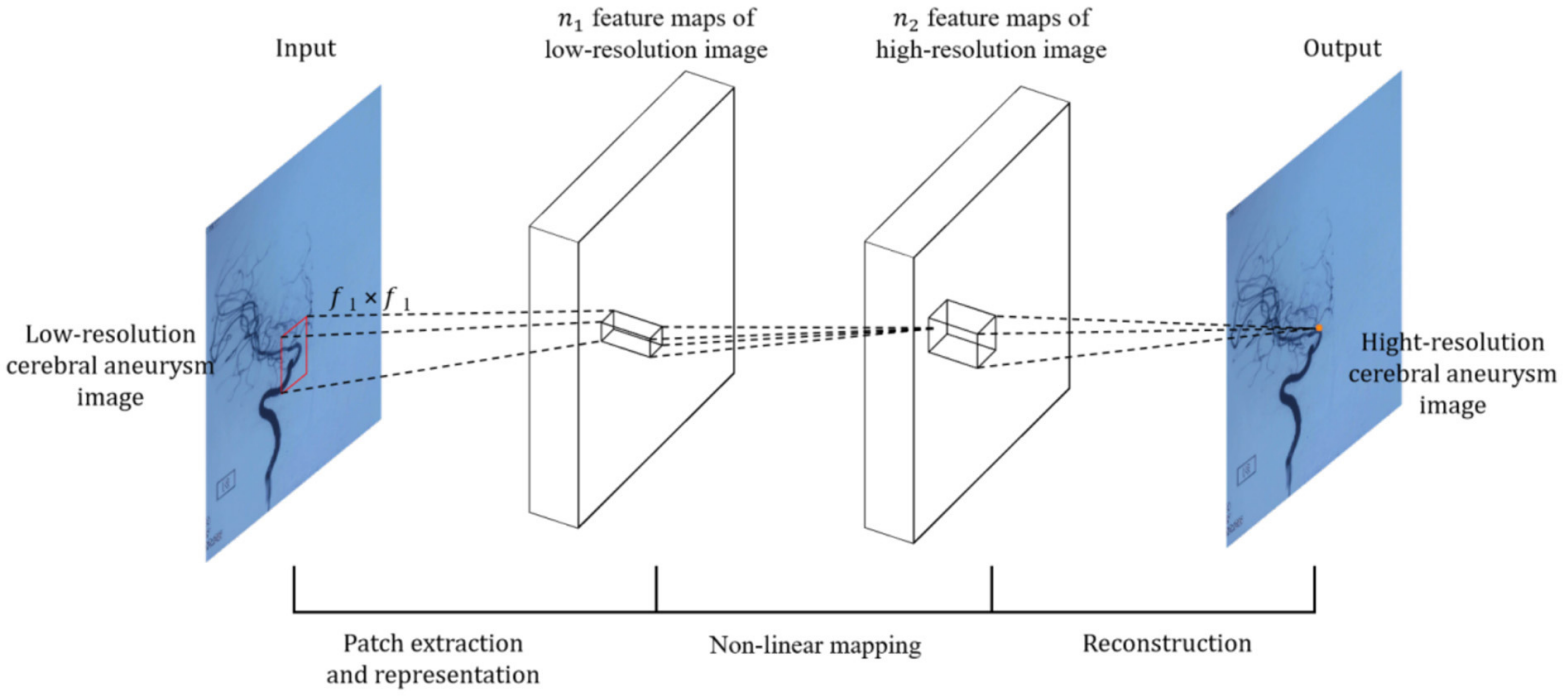
Schematic diagram of SRCNN neural network.

For the input image, SRCNN first needs to preprocess the input L.R. image, explicitly using the bicubic interpolation algorithm (Bicubic) to enlarge the low-resolution (L.R.) image to the size of the target image (20). The image is then fed into the deep learning network. Because it is an early application, the entire network structure has only three layers of convolutional neural networks, as shown in Figure 2.

Specifically, according to the theoretical knowledge of sparse coding, the author divides the overall network architecture into three modules: the underlying feature extraction module, non-linear learning, and reconstruction module (20).

(1) Feature extraction and representation: extract a low-resolution image patch from the L.R. image Y, and use a high-dimensional vector to represent an image patch. These vectors are combined to form a set of low-resolution feature maps, the number of which is equal to the dimension of the vector. The operations of the first layer are expressed, as shown by Equation (3).


(3) $$\begin{array}{l} {\text{F}}_{1}\left(\text{Y}\right)=\text{max}\left(0,{\text{W}}_{1}\times \text{Y}+{\text{B}}_{1}\right) \end{array}$$

where *W*_1_ and *B*_1_ represents the filter and offset, *W*_1_ the size is, *c* is the number of channels in the input image, is the size of the *n*_1_ filter, the number of filters, so the output *n*_1_ feature map *B*_1_ is one *n*_1_-dimensional vector.

(2) Non-linear mapping: Non-linearly map each high-dimensional vector to another high-dimensional vector. The high-dimensional mapping vector obtained through the mapping relationship can represent a high-resolution image block. These vectors are combined to form a feature map, and the operation of the second layer is expressed, as shown in Equation (4).


(4) $$\begin{array}{l} {\text{F}}_{2}\left(\text{Y}\right)=\text{max}\left(0,{\text{W}}_{2}\times {\text{F}}_{1}\left(\text{Y}\right)+{\text{B}}_{2}\right) \end{array}$$

where *W*_2_ dimension is, *B*_2_ is one *n*_2_-dimensional vector. *F*_2_ Represents the output *n*_2_ dimensional high-resolution image patch that will be used for reconstruction.

(3) Reconstruction: The high-resolution image patches are aggregated together to reconstruct the final high-resolution image, similar to the original high-resolution image. The last convolutional layer produces the final high-resolution image, defined as, as shown in Equation (5):


(5) $$\begin{array}{l} \text{F}\left(\text{Y}\right)={\text{W}}_{3}\times {\text{F}}_{2}\left(\text{Y}\right)+{\text{B}}_{3} \end{array}$$

where *W*_3_ dimension is, *B*_3_ is one *c*-dimensional vector.

The related literature shows that, compared with the state-of-the-art learning-based methods, the SRCNN model has a simple structure, high robustness, provides high accuracy, and restores images of high quality, indicating that convolutional neural networks can be applied to image classification involving computer vision for object recognition. The SRCNN method proposed by the authors learns the end-to-end mapping between low-resolution and high-resolution images with little additional pre-and post-processing other than optimization.

## Materials and methods

### Dataset

A total of 865 patients with unruptured saccular aneurysm (positive cerebral aneurysm) and 120 normal subjects without aneurysm who underwent routine physical examination (physical examination) or visited a doctor from January 2013 to May 2022 at Department of neurosurgery, the second affiliated of Guizhou Medical University Hospital were retrospectively collected (cerebral aneurysm negative) CTA image. After screening by inclusion and exclusion criteria, 500 subjects were included in the study, including 400 cerebral aneurysms positive and 100 cerebral aneurysms negative. The CTA dataset was randomly divided into a training group (*n* = 300, including 240 cerebral aneurysm positive cases and 60 cerebral aneurysm negative cases) and a testing group (*n* = 100, including 80 cerebral aneurysm positive cases and 20 cerebral aneurysm negative cases) according to the number of subjects. The ratio is 8:2.

See Table 1 for details.

**Table 1:** The usage of the dataset selected for the experiment.

	**Total**	**Training group *n* = 300**	**Test group *n* = 100**	**Proportion**
An unruptured cerebral aneurysm (positive)	400	240	80	8
Normal (negative)	100	60	20	2

The development of diagnostic criteria for a cerebral aneurysm is divided into “gold standard” and “silver standard” (21):

① The “gold standard” refers to the diagnosis of cerebral aneurysm patients by DSA;
② “Silver standard” refers to some patients whom DSA has not confirmed. Two radiologists with more than 5 years of experience review the original and reconstructed images, imaging reports and clinical history, and successive examinations (including CTA and MRA) to determine the most likely label for the diagnostic criteria. In the event of disagreement, a third deputy chief physician assists, and three radiologists vote to determine the presence of an aneurysm.

Inclusion criteria:

(1) Unruptured saccular aneurysm;
(2) No history of surgical clipping or coil embolization.

Exclusion criteria:

(1) subarachnoid hemorrhage;
(2) Severe stenosis of the cerebral atherosclerotic lumen;
(3) Poor image quality with noticeable artifacts;
(4) Other lesions in the brain, such as a tumor, intracranial hematoma, arteriovenous malformation, etc.

### ResNet architecture and model training

From a theoretical point of view, the deeper the network, the more sufficient the features extracted by the network, and the more favorable it is for image reconstruction (22). However, blindly increasing the depth will cause the gradient to disappear and the gradient to explore. In backpropagation, the gradient needs to be calculated; according to the chain derivation rule, a series of gradients are multiplied and then added. The obtained partial derivative updates the weight, and the update Equation (6) of the weight is as follows (23):


(6) $$\begin{array}{l} \text{w}=\text{w}-\alpha \frac{\partial \text{loss}}{\partial \text{w}} \end{array}$$

Where α is the learning rate. When the number of network layers is deepened, and the value of the gradient is <1, it can be seen from Equation 6 that the multiplication of multiple values <1 causes $\frac{\partial loss}{\partial }\;$the value to approach 0. At this time, the shallow weights of the network cannot be updated, resulting in insufficient training of the network; that is, the gradient disappears; on the contrary, if the value of the gradient is >1, the multiplication of multiple values >1 will result *in ∂loss*/∂*w* in an infinite value, resulting in the weight of It cannot be calculated, that is, the gradient explosion phenomenon occurs (24).

**Figure 3:**
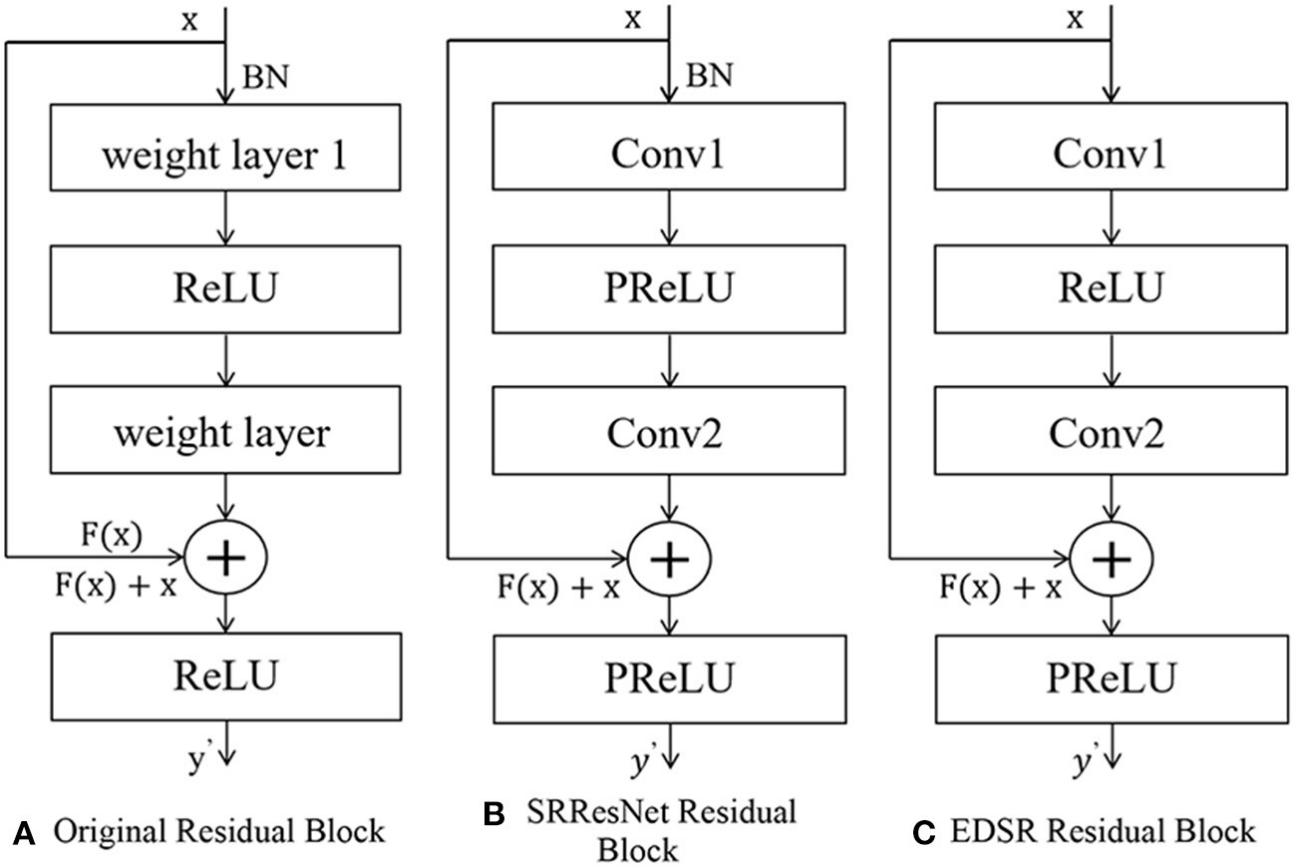
The structure of residual net. **(A)** Original residual block. **(B)** SRResNet residual block. **(C)** EDSR residual block.

The idea of residuals can effectively solve the above gradient problem. The residual structure allows the network to not degenerate with depth by stacking identity mapping layers on an external network (25). The residual block structure consists of a convolutional layer-batch normalization layer and an activation function layer, as shown in Figure 3. The residual structure introduces a fast connection based on the original network, and x is used as the input. After convolution transformation, the output *H*(*x*)becomes, at that time, which can ensure that the network forms an identity mapping; at that time, the network was regular. *F*(*x*) ≠ 0 Iterating, the weights are updated accordingly.

**Figure 4:**
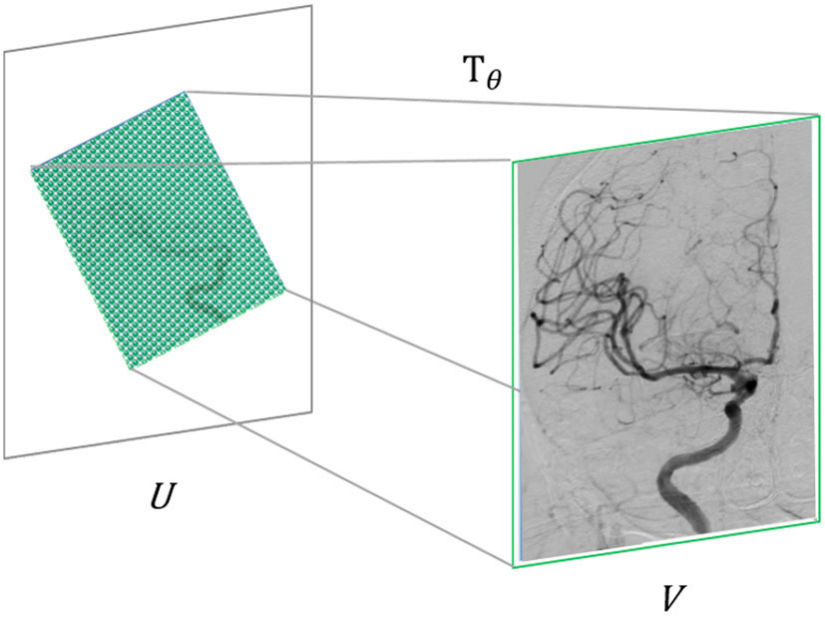
Schematic diagram of the attention mechanism in the spatial domain.

**Figure 5:**
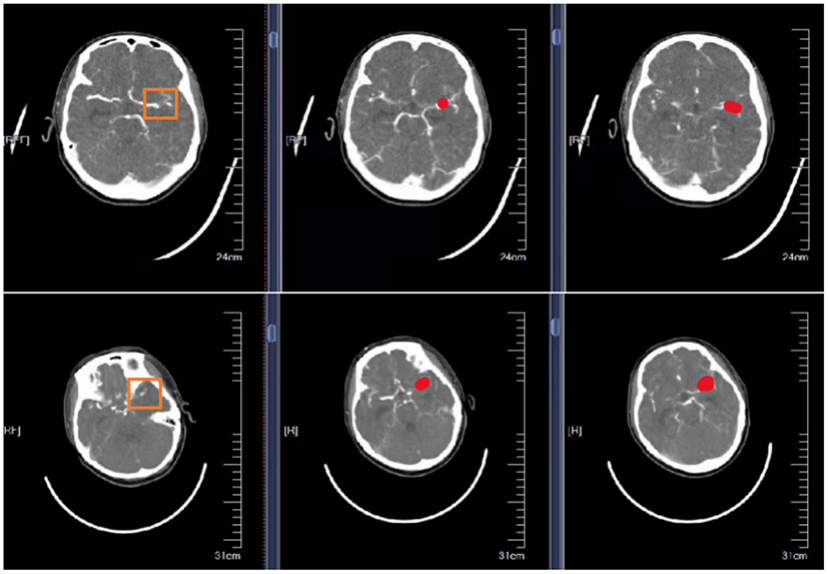
Schematic representation of correctly segmented cerebral aneurysms at different locations. The first column is the original CTA image. The second column is the result of physician annotation. The third column is the result of physician detection after high-resolution image reconstruction.

The generation part of SRGAN, SRResNet, uses 16 residual blocks to cascade to complete feature extraction. The structure of residual blocks is shown in Figures 2–**5B**. Each residual block contains two 3 × 3 convolutional layers. After layering, the B.N. layer and the PReLU activation function layer are connected to realize the fusion of low-frequency and high-frequency features. The reconstruction part can obtain a feature map containing rich, detailed information (26).

The EDSR model fine-tunes and improves the interior of the residual structure, and removes the B.N. layer, as shown in Figures 2–**5C**. The B.N. layer was initially used for high-level computer vision problems such as classification tasks, which can effectively implement the process of regular training and avoid overfitting (27). B.N. plays a significant role in classification tasks because image classification does not need to preserve image contrast and can use structural information to complete classification (28). The B.N. layer returns the mean value to 0 and the variance to 1. Although the absolute difference of pixels is ignored, it does not affect the classification task. For image super-resolution reconstruction, the B.N. layer will change the original contrast of the image and normalize the image's color distribution, which affects the reconstruction quality.

To sum up, removing the residual block structure of the B.N. layer has the following advantages. First, it can reduce memory usage and thus speed up the running time; the limit of normalization to 1 meets the requirements of the image in terms of color, contrast, and brightness.

### Self-attention mechanism

The attention mechanism is another effective means to improve the performance of neural networks (29). Its principle is mainly based on the attention distribution method of the human brain when processing signals. When the brain receives external signals, it tends to focus on what it is interested in to achieve efficient information extraction. In recent years, researchers have achieved many results by applying attention mechanisms to natural language processing, speech recognition, and image recognition (30).

In computer vision, the specific implementation of attention mechanisms is mainly divided into two categories: spatial domain and channel domain. The spatial domain attention mechanism refers to focusing the computational resources of the network on the spatial locations of interest in the image (31). Firstly, the spatial transformation network obtained by training *T*_θ_ extracts the area that needs attention from the input image U and, at the same time, uses the learned perspective transformation matrix to adjust it to a more easily discernible shape, thereby realizing computing resources. Reasonable distribution in the input image. The spatial domain attention mechanism can improve image recognition accuracy to a certain extent. In the super-resolution problem studied in this paper, it is usually necessary to take the complete image as the research object. For the fidelity of information, the image is generally not perspective. In transformation, we adopt the channel domain attention mechanism in this paper, as shown in Figure 4.

The principle of the channel domain attention mechanism is straightforward. A natural image usually consists of three channels of RGB or YCbCr. The importance of information in each channel must be unequal (32). The brain will focus on the information of specific channels, and relatively the remaining channels are ignored. Similarly, in the output feature map of the convolutional layer, the features of different channels are extracted by different convolution kernels, and their importance is obviously different, so they should be treated differently. Based on the above considerations, Hu et al. proposed the S.E. module (Squeeze-and-Excitation module) to simulate the channel attention mechanism (33). In the S.E. module, *W* × *H* the feature map with the size and number of channels as C is used as input. First, the squeeze operation is performed on each channel of the feature map, as shown in Equation (7):


(7) $$\begin{array}{l} {\text{z}}_{\text{e}}={\text{F}}_{\text{sq}}\left({\text{u}}_{\text{e}}\right)=\frac{1}{\text{W }\times \text{ H}}\overset{\text{H}}{\underset{\text{i}=1}{\sum }}\overset{\text{W}}{\underset{\text{j}=1}{\sum }}{\text{u}}_{\text{e}}\left(\text{i},\text{j}\right) \end{array}$$

The squeezing function *F*_*sq*_(·) does a global average pooling operation, encoding the spatial features on each channel into a global feature.

The squeeze operation yields a global description feature, followed by an excitation operation to learn the importance of different channel features. In the S.E. module, this operation is implemented by two fully connected layers, and the specific operation process is shown in Equation (8):


(8) $$\begin{array}{l} \text{s}={\text{F}}_{\text{ex}}\left(\text{z}\right)=\sigma \left({\text{W}}_{2}\times \text{ReLU}\left({\text{W}}_{1}\times \text{z}\right)\right) \end{array}$$

where σ(·) represents the sigmoid activation function; *ReLU*(·) represents the ReLU activation function; *W*_1_ and *W*_2_ represents the weight coefficients of the two fully connected layers, respectively, r is a dimension reduction coefficient, so the first fully connected layer also plays a role in dimension reduction. Then the second fully connected layer, the connection layer restores the original dimensionality, which ensures that the entire S.E. module has only a small number of parameters.

Finally, the channel attention assignment is completed by assigning the learned activation value as a weight to each channel on the original feature map.

### Performance evaluation indicator

Image Quality Assessment (IQA) for the reconstructed image is also an essential field in super-resolution reconstruction technology (34). In order to make the reconstructed image or video as close to the actual H.R. image as possible, a reasonable image or video quality evaluation index is required. Generally speaking, the image quality criterion usually includes subjective and objective evaluations. The two indicators are described in detail below.

#### Subjective evaluation criteria

The subjective evaluation method is a method in which the observer subjectively evaluates the quality of the reconstructed image and gives the corresponding score through the analysis of people's eyes (35). Commonly used methods include the mean subjective score (Mean Opinion Score, MOS) or the mean subjective score difference (Differential Mean Opinion Score, DMOS). The MOS method is proposed by the SRGAN paper and has a good evaluation effect. Generally, the test images are directly handed over to the testers, and the images are scored according to their personal feelings. The scores are divided into five grades, corresponding to 1–5 points. The better the effect, the higher the score, which can be expressed by the following Equation (9):


(9) $$\begin{array}{l} \text{MOS}=\frac{\sum_{\text{i}=1}^{\text{N}}{\text{R}}_{\text{i}}}{\text{N}} \end{array}$$

Where R represents the score, N represents the number of people tested, and the average is taken. It can be seen from the Equation that the higher the mos score, the better. For the subjective evaluation of reconstructed images, people are the main body. It is more in line with human subjective visual perception and more in line with the fact that people are often the foremost observers of images. However, this method also has shortcomings. First, the evaluation standard depends on the person, and there is no mathematical principle as the basis for evaluation. Therefore, many repeated experiments are often carried out. Secondly, the subjective evaluation relies on human vision and is also affected by the surrounding environment. Influence, such as light, angle, etc., has certain limitations, so the experimental environment. Therefore, it takes a long time, and many workforce and financial resources are invested. The difficulty of operation is relatively great. Therefore, this subjective evaluation method using MOS is rarely used. More is to observe through the human eye or zoom in on a particular area and then observe and compare. Therefore, the objective evaluation method is the mainstream of the current evaluation method.

#### Objective evaluation criteria

The method of objectively evaluating the quality of the reconstructed image generally uses a mathematical model to give a specific quantitative value, which is separated from the judgment of human subjective consciousness and has a specific mathematical theoretical basis. It starts from an objective mathematical model, separates from the judgment of people's subjective consciousness, and defines the standard through mathematical function Equations (36). The reconstructed images to be evaluated have the same evaluation criteria, and there is no difference in subjective evaluation criteria. So it has always been the focus of IQA research. Objective IQA methods are divided into three categories: full-reference methods that use reference images for evaluation, such as peak signal-to-noise ratio (PSNR) (37). Pixel-wise loss for PSNR calculation, and MSE in addition to that. Both calculations are essentially the same. PSN calculates the ratio through 1og. The larger the value, the better, indicating better reconstruction quality. The smaller the MSE, the better. In addition, there is structural similarity (Structure Similarity, SSIM). Based on the partial reference method of extracting feature comparison, the focus and difficulty of partial image reference are to find suitable feature information; no reference method without a reference image (i.e., blind IQA), such as mean, standard deviation, etc.

(1) Mean Square Error (MSE), the Equation is shown in Equation (10).


(10) $$\begin{array}{l} \text{MSE}=\frac{1}{\text{M }\times \text{ N}}\overset{\text{M}}{\underset{\text{i}=1}{\sum }}\overset{\text{N}}{\underset{\text{j}=1}{\sum }}{\left({\text{f}}^{\prime }\left(\text{i},\text{j}\right)-\text{f}\left(\text{i},\text{j}\right)\right)}^{2} \end{array}$$

It represents the expectation of the sum of squared losses per pixel between the reconstructed image and the actual H.R. image. *f*′ *Where F*(*i, j*) and *f*(*i, j*) represents the image to be evaluated and the original image, respectively, M, N represents the length and width of the image, respectively. MSE, in terms of super-resolution, is the expected value of the square of the difference between the pixel estimated value and the actual image pixel value. The smaller the MSE value, indicating that it is closer to each pixel of the image and the reconstruction quality is better. The restoration degree is good; the information loss is slightly closer to the HE image.

(2) Peak-Signal to Noise Ratio (PSNR) (38), as the name implies, calculates a ratio. Because of the similarities between PSNR and MSE in some aspects. We can think of it as the logarithmic representation of MSE. Define Equation (11) as follows:


(11) $$\begin{array}{l} \text{PSNR}=10\frac{{\text{Maxvalue}}^{2}}{\text{MSE}}=10\frac{2^{\text{bits}}\;-\;1}{\text{MSE}} \end{array}$$

The larger the PSNR value, the better the quality of the reconstructed image. The *Maxvalue*value is 255 because the pixels in the image are stored quantized in the computer; each pixel occupies 8 bits, and the computer is binary, so calculate 2 to the eighth power and subtract one to get 255. The algorithm is simple, and the inspection speed is fast. However, based on the error between the corresponding pixel points, the presented difference value is not proportional to the subjective human feeling. However, the pure pursuit of the point-to-point loss between pixels ignores human beings' main body of visual evaluation.

## Experiments and analysis

### Experimental details

The operating system used in this experiment is the Ubuntu 16.06 operating system based on the Linux system. The deep learning framework that implements the neural network is Tensorflow, version 2.0. The programming language is Python3.6, and the graphics card is 1080Ti. The compilation environment is pycharm and juypter notebook. There are also some shared third-party libraries, such as Open CVnumpy and so on.

The deep learning model optimization algorithm adopts the Adam optimization algorithm. Adamy optimization algorithm is one of the most popular optimization algorithms out there. The learning rate can be adjusted adaptively. The learning rate of each training has a fixed range, and the parameter changes are relatively small. The advantage of Adagrad is that it is suitable for dealing with sparse gradients but not suitable for dealing with non-stationary targets. The Adam optimization algorithm has improved this point. Excellent results are achieved very quickly. The Equation is shown in Equations (12)–(16) (39, 40):


(12) $$\begin{array}{l} {\text{m}}_{\text{t}}=\beta_{1}{\text{m}}_{\text{t}-1}+\left(1-\beta_{1}\right){\text{g}}_{\text{t}} \end{array}$$

(13) $$\begin{array}{l} {\text{v}}_{\text{t}}=\beta_{2}{\text{v}}_{\text{t}-1}+\left(1-\beta_{2}\right){\text{g}}_{\text{t}}^{2} \end{array}$$

(14) $$\begin{array}{l} \hat{{\text{m}}_{\text{t}}}=\frac{{\text{m}}_{\text{t}}}{1-\beta_{1}^{\text{t}}} \end{array}$$

(15) $$\begin{array}{l} \hat{{\text{v}}_{\text{t}}}=\frac{{\text{v}}_{\text{t}}}{1-\beta_{2}^{\text{t}}} \end{array}$$

(16) $$\begin{array}{l} \theta_{\text{t}+1}=\theta_{\text{t}}-\frac{\eta }{\hat{{\text{v}}_{\text{t}}}+\varepsilon }\hat{{\text{m}}_{\text{t}}} \end{array}$$

Specific parameters use the default parameters; The learning rate is a 1 × 10^−4^ value εto 10^−8^ prevent the divisor from being 0. The data is trained after data augmentation. Because of the need to initialize the weights of the neural network. We use the He initialization strategy to initialize the network. The mathematical form is shown in Equation (17):


(17) $$\begin{array}{l} \text{W}\sim \text{N}\left(0,\sqrt{\frac{2}{{\text{n}}_{1}}}\right) \end{array}$$

Where *N* represents the uniform distribution and *n*_1_ represents the first layer of the neural network, through this initialization method, the initial weight can be prevented from being too large or too small. It prevents the network from having gradient dispersion (gradient disappearance) and other difficult-to-train problems during training.

### Experimental metrics and results

#### Objective evaluation

In the following, we compare and analyze our proposed algorithm and other super-resolution algorithms from an objective evaluation method. We use two evaluation indicators as an objective evaluation method: peak signal-to-noise ratio and structural similarity. PSNR and SSIM measure the reconstructed E image, and the larger the value, the better. The specific calculation method and meaning are described in the fourth subsection of paper 2. I will not go into details here.

For the test set, we divided it into two parts, corresponding to abdominal gastric adenocarcinoma images and abdominal esophageal cancer C.T. images, respectively. Under the 2× magnification factor and 4× magnification factor, the PSNR and SSIM corresponding to the test set are counted. The corresponding average value is taken as the final objective result of the test set. The experimental results are shown in Tables 2, 3 below.

**Table 2:** PSNR experimental data.

**Data set**	**Scale**	**Bicubic**	**SRCNN**	**ESPN**	**FSRCNN**	**Our method**
Brain aneurysm CTA imaging	2	28.54	30.65	32.01	31.91	33.01
Brain aneurysm CTA imaging	4	24.55	25.52	26.93	26.69	28.39

**Table 3:** SSIM experimental data.

**Data set**	**Scale**	**Bicubic**	**SRCNN**	**ESPN**	**FSRCNN**	**Our method**
Brain aneurysm CTA imaging	2	0.9400	0.9301	0.9324	0.9341	0.9521
Brain aneurysm CTA imaging	4	0.8653	0.8801	0.9023	0.8879	0.9138

According to the above two tables, the objective evaluation indicators PSNR and SSIM show the worst effect of bicubic interpolation, which is also in line with our subjective visual evaluation. The super-resolution C.T. images reconstructed by the SRCNN, ESPCN, and FSRCNN models are on the C.T. image test set. Compared with the traditional interpolation algorithm, it has been dramatically improved. At the same time, our proposed deep learning model has achieved the corresponding improvement based on the former. The PSNR is improved by about 5 dB compared with the traditional bicubic interpolation algorithm and about 1 dB compared with the previous deep learning model. On the SSIM indicator, it is about 0.1–0.3 higher than other S.R. models. At the same time, in terms of horizontal comparison, the PSNR and SSIM of the image enlarged by 2. times are better than the C.T. image reconstructed by 4 times. Explain that small magnifications of images are better for training during reconstruction. If the magnification factor is too significant, the reconstruction effect will be worse because of the increased information to be learned.

Therefore, summarizing the above subjective evaluation methods and objective evaluation indicators and comparing them with the traditional double interpolation algorithm and the classic deep learning S.B. model fully demonstrates the superiority of our proposed algorithm for super-resolution reconstruction on abdominal C.T. medical images, sex and effectiveness.

#### Residual network model effect

① The 3D U3-Net model missed 36 aneurysms, including 22 in ICA, 4 in MCA, 7 in ACA, 2 in PCA, and 1 in B.A. The diameters of missed aneurysms ranged from 2.20 to 5.38 mm. ② 37 false-positive aneurysms, 9 from cerebral aneurysm-negative subjects and 28 from cerebral aneurysm-positive subjects, of which 13 were located in the ICA, 12 were located in orbit, 4 were located in the ethmoid sinus, and 6 were located in the neck Veins, 1 in the ACA and 1 in the left FCA. ③ The model found two newly missed aneurysms, one in the left MCA and one in the left VA (40).

Are shown in Figure 5 schematic illustration of correctly classified aneurysms at different locations.

#### Residual network model performance

The diagnostic sensitivity of the 3D U2-Net model for aneurysms with diameters >3 mm was 85.9% (171/199), which was higher than that for aneurysms <3 mm in diameter (23/36, 63.7%). The difference was statistically significant. Significance (*P* = 0.001 < 0.05). The diagnostic sensitivity of the 3D U-Net model in different locations of the aneurysm (anterior circulation and posterior circulation), different field strengths (1.5 and 3.0 T), and different imaging equipment (Philips, Siemens, GE) had no statistically significant difference (*P* > 0.05), see Table 4 for details.

**Table 4:** Model checking performance.

**Parameters**	**Test set**
Number of examiners	268
Number of aneurysms	235
Sensitivity	82.7% (194/235)
Positive predictive value	82.3% (194/236)
Specificity	86.0% (43/50)
Aneurysm size	Sensitivity
<3 mm	64.7% (23/36)
3–7 mm	80.2% (95/118)
>7 mm	93.3% (76/81)
**Aneurysm location**	
ICE	82.8% (130/157)
MCA	70.3% (19/27)
ACA	74.3% (29/39)
PCA	40.0% (2/5)
BA	91.6% (11/12)
VA	100% (3/3)

#### Comparison of aneurysm detection

The 3D U2-Net model is compared with the indicators detected by radiologists of different seniority, as shown in Table 5. 3DU-Net model detected a total of 236 aneurysms, Physician 1 and Physician 2 217 and 225 were detected, respectively. The sensitivities of the three groups were Physician 1 < 3D U2-Net model < Physician 2, and the difference was not statistically significant (Physician 1 vs. model, *P* = 1.000; Physician 2 vs. model, *P* = 0.170), diagnostic specificity and positive predictive value were Physician 2 > Physician 1 > 3DU3-Net model, the false positive rate of 3D TU-Net model was 0.14 cases/case, which was higher than that of Physician 1 (0.07 cases/case) and Physician 2 (0.06 cases/case). The performance of the three groups in different aneurysm sizes, locations, field strengths and the sensitivity of imaging equipment are shown in Table 5.

**Table 5:** Comparison of physician detection and model-assisted physician detection results.

**Diagnosis technique**	**Check out(s)**	**Missed/misdiagnosed (pcs)**	**Sensitivity (%)**	**Positive predictive value (%)**	**Specificity (%)**	**False-positive rate (pieces/example)**
Physician 1	217	37, 19	84.3	91.2	90.0%	0.07
Model aided	236	36, 37	84.7	84.3	88.0%	0.14

## Discussion

### Review of the experimental procedure

This paper is mainly based on the multi-scale joint convolutional neural network proposed in Section Materials and methods. In order to stack more networks and enhance the flow of feature information between networks, a residual network is introduced. Besides, in the existing research, most S.R. models on medical images use one-shot reconstruction and generalize the problem of poor image reconstruction quality for prominent magnification factors. Considering that it becomes challenging to reconstruct the image due to the increased loss of information under prominent magnification factors, a multi-level output network is introduced to achieve progressive reconstruction, and our framework has a certain flexibility to obtain intermediate-level outputs; it is realized that the same network can be reconstructed with different multiples. Experiments are carried out on abdominal C.T. medical images. Experiments are carried out on CTA medical images of the brain. The experimental results show that the improved network proposed by us can enhance the quality of the reconstructed images under large magnification and achieve better results than the bicubic interpolation algorithm and the ESPCN algorithm. Moreover, CTA images of cerebral aneurysms verify that our model has good generality and generalization.

### Comparison with other studies

According to the above two tables, the objective evaluation indicators PSNR and SSIM show the worst effect of bicubic interpolation, which is also in line with our subjective visual evaluation. The super-resolution C.T. images reconstructed by the SRCNN, ESPCN, and FSRCNN models are on the C.T. image test set. Compared with the traditional interpolation algorithm, it has been dramatically improved. At the same time, our proposed deep learning model has achieved the corresponding improvement based on the former. The PSNR is improved by about 5 dB compared with the traditional bicubic interpolation algorithm and about 1 dB compared with the previous deep learning model. On the SSIM indicator, it is about 0.1–0.3 higher than other S.R. models. At the same time, in terms of horizontal comparison, the PSNR and SSIM of the image enlarged by 2. times are better than the C.T. image reconstructed by 4 times. Explain that small magnifications of images are better for training during reconstruction. If the magnification factor is too significant, the reconstruction effect will be worse because of the increased information to be learned.

Therefore, summarizing the above subjective evaluation methods and objective evaluation indicators and comparing them with the traditional double interpolation algorithm and the classic deep learning S.B. model fully demonstrates the superiority of our proposed algorithm for super-resolution reconstruction on abdominal C.T. medical images, sex and effectiveness.

### Innovations

Based on the field of medical images, this paper focuses on the super-resolution reconstruction technology of medical images, from the traditional super-resolution reconstruction technology—based on interpolation, based on sparse dictionaries and other methods, and is composed of deep learning widely used in various fields. The convolutional neural network, one of the units, is introduced, focusing on the deep learning-based super-resolution reconstruction of several typical S.B. models, SRCNN, ESPCN, etc. Given the shortcomings of the traditional deep learning S-up model in super-resolution medical image reconstruction, a summary is made, corresponding improvements are made in response to the problems, and two deep learning models are proposed for abdominal C.T. medical images. Super-resolution reconstruction. The innovation points of this study are summarized as follows:

(1) In the existing research, most deep learning models based on medical image super-resolution reconstruction use a single-scale convolution kernel to build the network, ignoring the difference in the information contained in images of different scales and feelings wild information. In order to fully extract multiple different scale information, we propose a multi-scale joint convolutional neural network for super-resolution reconstruction in the field of C.T. medical images.
(2) Introduce local residual learning to form a multi-scale joint residual block, which is used as the basic unit to avoid the gradient disappearance and gradient explosion problems caused by the deepening of the network layer. In addition, both SRCNN, ESPCN and other networks perform single-multiple L.R. image reconstruction and cannot perform L.R. image reconstruction output with multiple magnifications simultaneously, poor quality. Therefore, in response to this problem, the idea of upsampling the Laplacian pyramid network is introduced, and a multi-level output network is constructed to achieve the same network, which can output multiple magnified images simultaneously. It realizes multi-level magnification and step-by-step reconstruction and enhances the quality of reconstructed images under prominent magnification factors.

### Research significance

Through the results of this study, it can be found that the deep learning CAD system based on 3D U-Net has the following advantages in the detection of cerebral aneurysms: First, it helps physicians to perform an initial screening of diseases, which can effectively assist radiologists in reducing work intensity and improving work efficiency; Second, the accuracy of identifying cerebral aneurysm is equivalent to that of a radiology resident, and the “human-machine integration” mode in clinical applications can significantly improve the accuracy of diagnosis; third, the standardized process of a CAD system can ensure the objectiveness of diagnostic results stability, repeatability, and the ability to learn and improve from feedback continuously. Note that extreme learning deep neural networks (41) can be implemented for the high-resolution medical image reconstruction of cerebral aneurysm in the future. The three dimensional visualization framework (38) in monitoring of cerebral aneurysm can be enhanced based on such advanced spatial models.

## Conclusion

Our study is a retrospective analysis. In order to further verify the clinical application value of ResNet in the detection of cerebral aneurysms, we next plan to conduct a prospective study based on multi-center clinical data. Moreover, our algorithm uses only CTA source images and no MIP images and using both source and MIP images may show better performance in detecting intracranial aneurysms. Finally, the algorithm was only developed for cystic unruptured aneurysms, and its performance in other types of aneurysms has not been studied. Fourth, the training dataset does not contain enough specific regions and sizes data. More unusual locations and small aneurysms should be added to the training process to improve the algorithm further.

## Data availability statement

The raw data supporting the conclusions of this article will be made available by the authors, without undue reservation.

## Ethics statement

Ethical review and approval was not required for the study on human participants in accordance with the local legislation and institutional requirements. Written informed consent for participation was not required for this study in accordance with the national legislation and the institutional requirements. Written informed consent was obtained from the individual(s) for the publication of any potentially identifiable images or data included in this article.

## Author contributions

All authors listed have made a substantial, direct, and intellectual contribution to the work and approved it for publication.

## Conflict of interest

The authors declare that the research was conducted in the absence of any commercial or financial relationships that could be construed as a potential conflict of interest.

## Publisher's note

All claims expressed in this article are solely those of the authors and do not necessarily represent those of their affiliated organizations, or those of the publisher, the editors and the reviewers. Any product that may be evaluated in this article, or claim that may be made by its manufacturer, is not guaranteed or endorsed by the publisher.
